# A Rare Presentation of a Spinal Lesion: Immunoglobulin G4-Related Hypertrophic Spinal Pachymeningitis

**DOI:** 10.7759/cureus.83120

**Published:** 2025-04-28

**Authors:** Azhar S Alshoumer, Basma M Alyamany, Lina T Alanazi

**Affiliations:** 1 Histopathology Department, King Fahad Medical City, Riyadh, SAU

**Keywords:** hypertrophic pachymeningitis., idiopathic pachymeningitis, igg4-related spinal pachymeningitis, immunoglobulin g4 (igg4)-related disease, spinal pachymeningitis

## Abstract

Immunoglobulin G4-related disease is a rare systemic condition that can affect multiple organs, though involvement of the central nervous system is uncommon. This report discusses an unusual case of a 64-year-old man who experienced neurological symptoms due to a spinal lesion. Imaging revealed a compressive mass at the cervical spine level, which was surgically removed and later confirmed to be IgG4-related hypertrophic spinal pachymeningitis. This case highlights the importance of considering rare inflammatory conditions in the differential diagnosis of spinal lesions and emphasizes the role of histopathological confirmation for accurate diagnosis and treatment planning.

## Introduction

Immunoglobulin G4-related disease (IgG4-RD) is a recently described immune-mediated chronic condition characterized by fibrosing and inflammatory processes that lead to the formation of tumefactive lesions [1]. It presents with a wide range of clinical manifestations and can involve multiple organs, often mimicking other conditions such as malignancies, infections, and other inflammatory disorders.

The pancreas, salivary glands, retroperitoneum, and lymph nodes are the most frequently affected sites [2-3]. Central nervous system involvement is rare, with most reported cases presenting as hypertrophic pachymeningitis (HP) or hypophysitis [4]. The hallmark histopathological features include a dense lymphoplasmacytic infiltrate rich in IgG4-positive plasma cells, obliterative phlebitis, and fibrosis [5-6].

Magnetic resonance imaging (MRI) is the imaging modality of choice for the initial diagnosis of an extra-axial mass or lesion. Dural thickening can be observed in a wide range of pathological conditions with various etiologies, including infectious diseases (e.g., syphilis, tuberculosis, sarcoidosis, fungal infections), neoplastic processes (e.g., meningioma, leptomeningeal carcinomatosis, lymphoma, melanoma), connective tissue disorders, storage diseases, prolonged dialysis, and extended administration of intrathecal medications [7].

Early recognition is essential, as the disease is generally treatable; however, advanced fibrotic changes may become irreversible. In this case report, we present a 64-year-old male who developed spinal cord compression due to an intradural extramedullary lesion, which was histologically confirmed to be IgG4-related spinal pachymeningitis.

## Case presentation

A 64-year-old gentleman presented with numbness and distal motor weakness; his symptoms started two months earlier. His medical history is remarkable for diabetes and dyslipidemia. He denied gait instability, facial asymmetry, or trauma.

Neurological examination revealed intact facial sensation. Motor strength was full (5/5) in all limbs except for right elbow flexion/extension (4+/5), finger abduction/adduction (4/5), and right ankle dorsiflexion (3/5). Sensory assessment showed decreased pinprick sensation in the right upper limb and increased sensation in the right lower limb, with no sensory level. Reflexes were brisk in the right lower limb with non-sustained clonus, upgoing plantar response, positive Romberg's sign, and negative Hoffman's sign.

The laboratory findings were within normal ranges, including infectious and inflammatory blood markers. The patient’s serum IgG4 was measured at 361.500 mg/L (normal range is 39.200-864.000 mg/L), which was within normal range.

**Figure 1:**
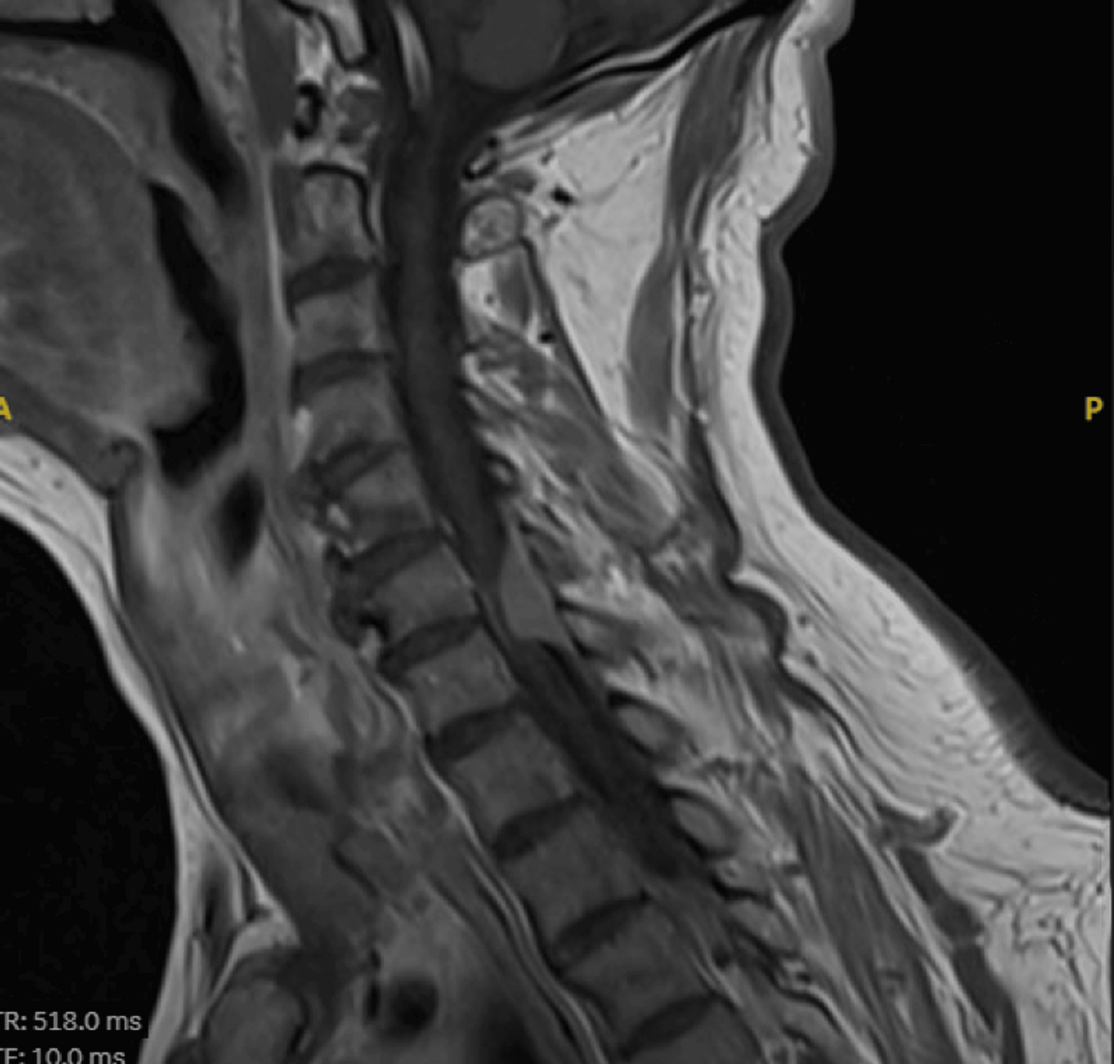
Preoperative cervical sagittal MRI of the patient demonstrated intradural extramedullary lesion at level of C6-C7 causing cord compression.

Spinal MRI revealed an intradural extramedullary enhancing lesion measuring 0.9 x 1.4 x 2.7 cm (AP TR CC). It was situated at the levels of the C6-C7 vertebrae, was hypointense on T2 and isointense on T1. It demonstrated avid post-contrast enhancement. The bulk of the lesion displaced the spinal cord to the left side, causing focal severe stenosis of the spinal cord (Figure 1).

Based on clinical and imaging findings, meningioma was considered the top differential diagnosis, and the patient underwent posterior cervical decompression and excision of the cervical mass. The surgical findings were consistent with the radiological impressions, revealing an extramedullary lesion measuring approximately 2.5 cm.

**Figure 2:**
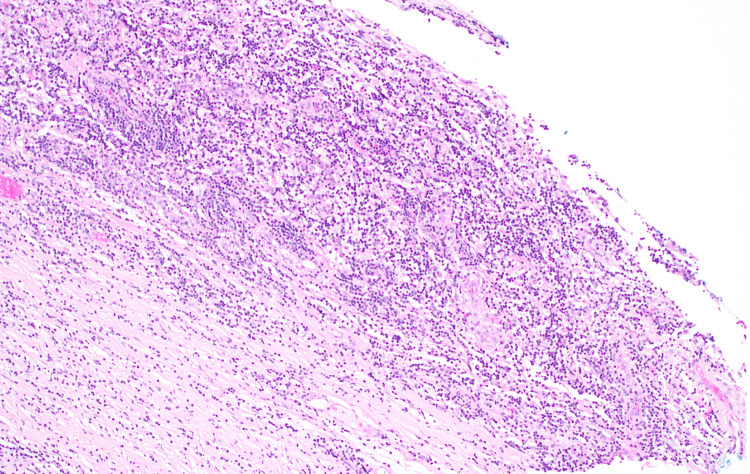
Photomicrograph (hematoxylin & eosin) shows characteristic features of immunoglobulin G4-related spinal pachymeningitis. Low-power view of a resected intradural lesion show marked lymphoplasmacytic cellular infiltrate with predominance of plasma cells in background of fibrous tissue.

**Figure 3:**
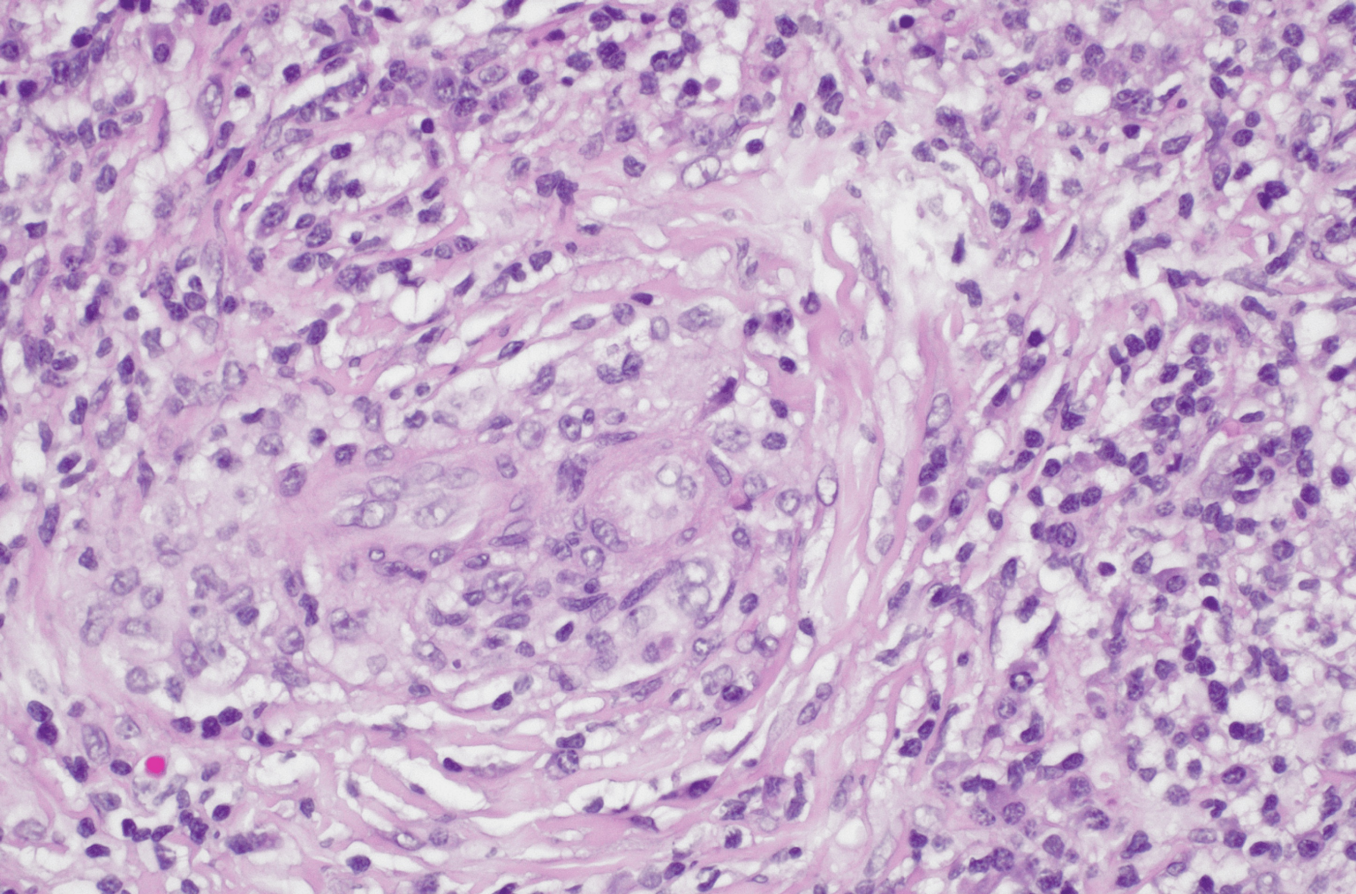
Photomicrograph (hematoxylin & eosin) shows characteristic features of immunoglobulin G4-related spinal pachymeningitis, including obliterative phlebitis. This view demonstrates the inflammatory cells attacking and destroying the wall of the blood vessel.

**Figure 4:**
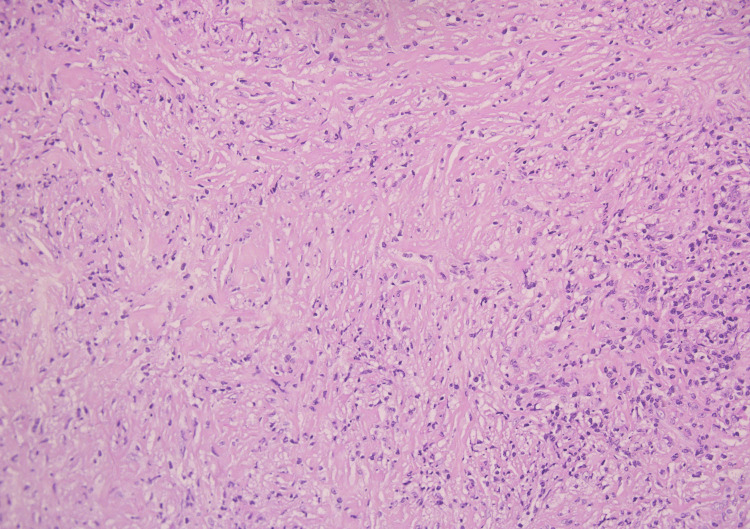
Photomicrograph (hematoxylin & eosin) shows characteristic features of immunoglobulin G4-related spinal pachymeningitis and diffuse storiform fibrosis.

The excised specimen was sent to our histopathology department as two fragments. The larger fragment measured 1.8 cm in its greatest dimension. It has a white-tan homogenous gross cut surface. The microscopic examination showed fibrotic tissue with marked lymphoplasmacytic cellular infiltrate with predominance of plasma cells (> 50 per high-power field (HPF)), areas of storiform fibrosis, and obliterative phlebitis were identified (Figures 2-4).

**Figure 5:**
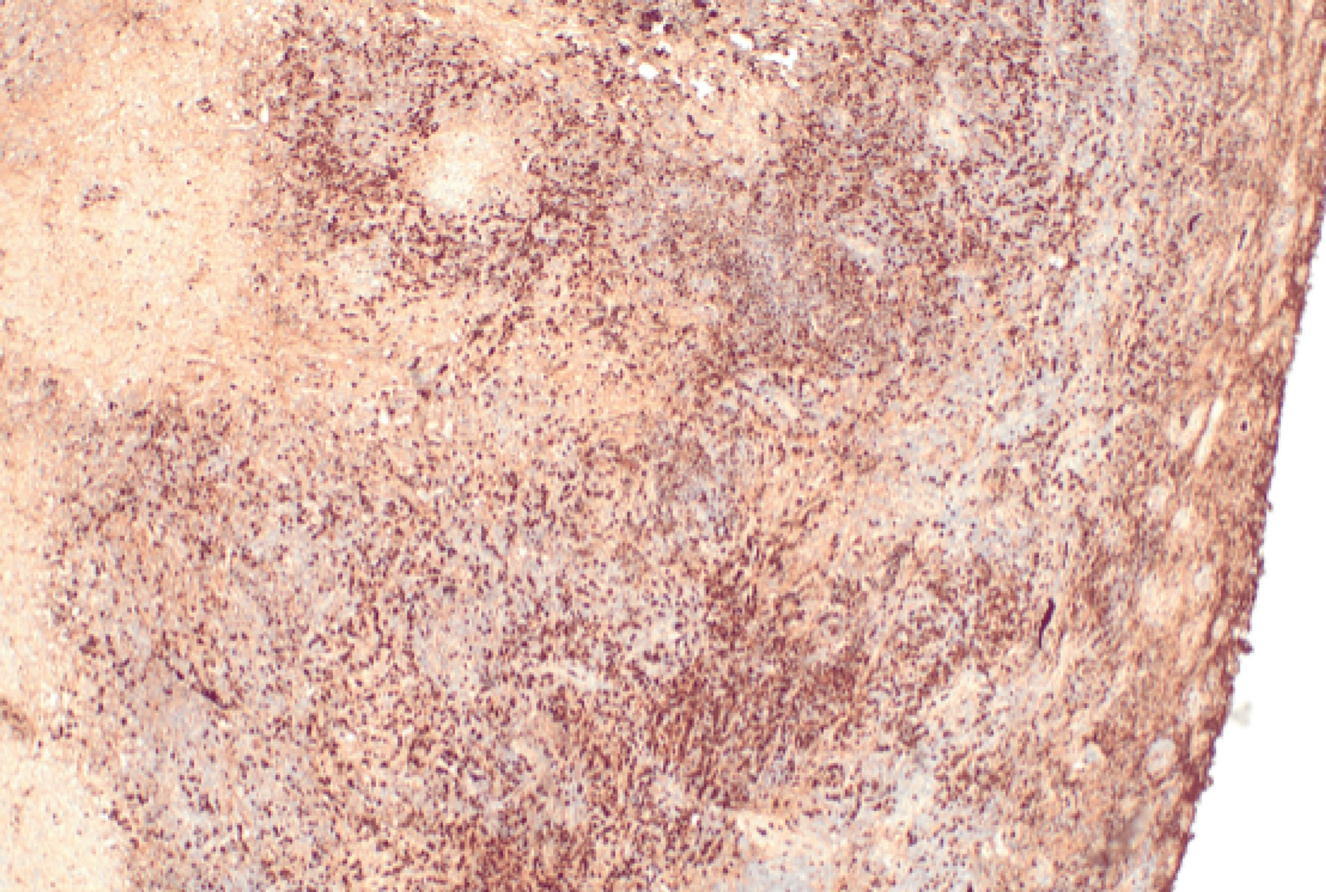
Photomicrograph of immunohistochemical stain shows IgG+: positive in plasma cells (> 50 per HPF). HPF: high-power field

**Figure 6:**
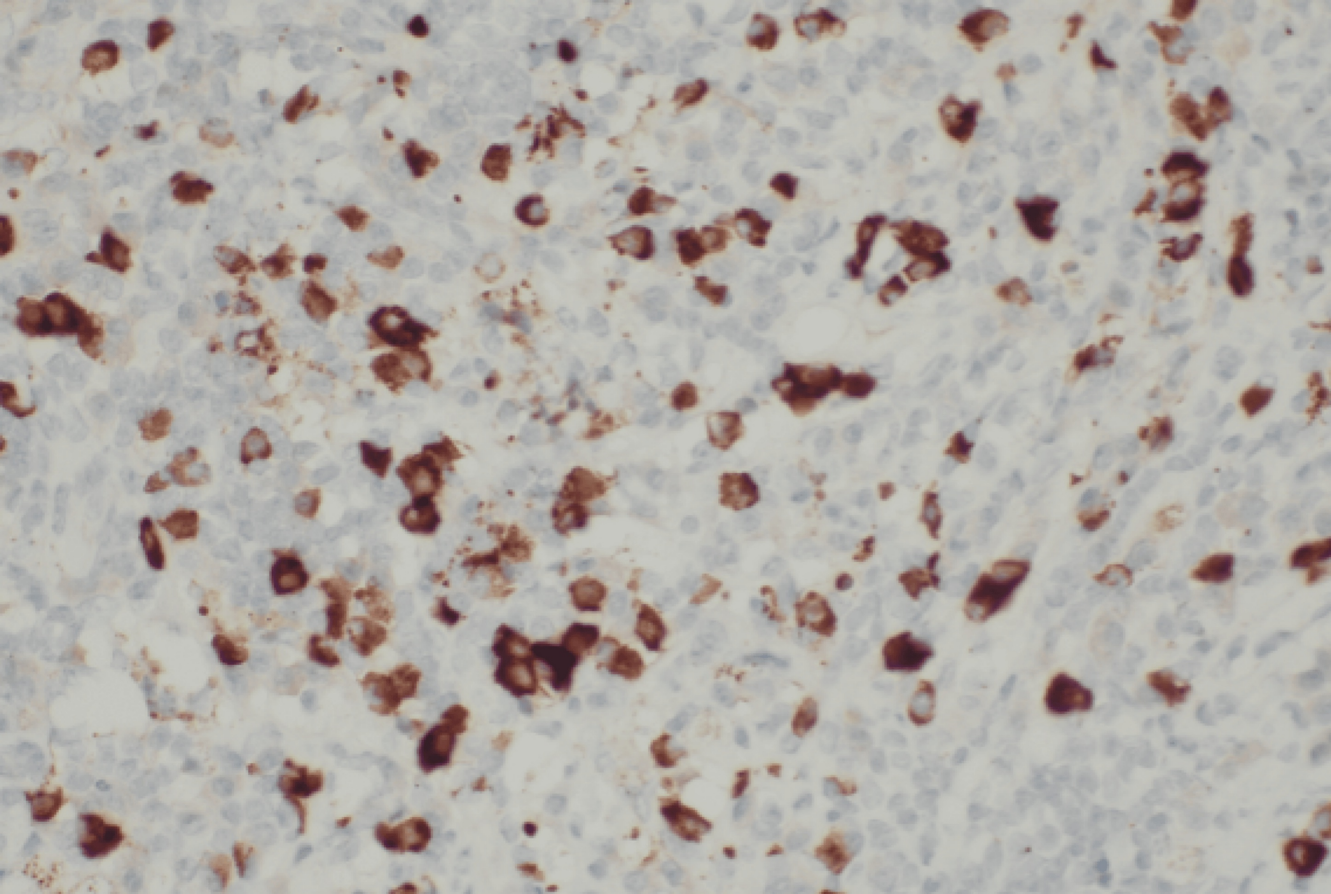
Photomicrograph of immunohistochemical stain shows IgG4+ is positive in plasma cells (> 10 per HPF). The ratio is our case IgG4+/IgG+ ratio: plasma cells >40%. HPF: high-power field

Immunohistochemical stains were performed to show that plasma cells are immunopositive for EMA, CD138, and IgG4 >10 per HPF (~50 positive plasma cells per HPF), with the IgG/IgG4 ratio being more than 40% (Figures 5-6). Ki67 proliferative index was low (~2-5%). Cells showed no restriction to Kappa or Lambda, and are immunonegative for progesterone receptor, S100, CD1a, CD30, or EBV. Special stains (PAS, GMS, and acid-fast bacilli) were negative for acid-fast bacilli and fungi. With these typical findings, the diagnosis of immunoglobulin G4-related spinal pachymeningitis was confirmed. The patient was treated with corticosteroids following the excision, which resulted in a positive response.

## Discussion

Meningeal involvement in IgG4-RD was later identified and first reported in 2009 [8]. Although the significance of IgG4 was first noted in 1961 in a patient with pancreatitis and elevated gamma globulins [9], its role as a diagnostic marker was not recognized until 2003, when increased IgG4 levels were reported in autoimmune pancreatitis [10].

HP is a fibroinflammatory condition characterized by thickening of the cranial or spinal dura mater [11]. It can present with a wide range of neurological symptoms, including cranial nerve palsies, venous thrombosis, stroke, intracranial hypertension, and sensorimotor deficits [11]. The disease is more prevalent in men, with a median age of onset of approximately 53 years. [11].

HP has diverse etiologies, including neoplasms, infections, trauma, medications, or it may occur idiopathically. Retrospective studies have identified that a significant proportion of idiopathic HP cases are attributable to IgG4-RD, suggesting a strong association between the two conditions [11].

IgG4-RD most commonly involves the lymph nodes, pancreas, gallbladder, eyes, kidneys, salivary glands, and retroperitoneum. However, it has the potential to affect nearly any organ, except the synovium [12,13].

The pathogenesis of HP associated with IgG4-RD is not fully understood, but it is hypothesized to involve an immune response to an unidentified antigen [14]. Unlike patients with orbital involvement, those with meningeal disease often lack widespread systemic symptoms. Grossly, affected organs may appear enlarged and indurated.

The disease is defined histologically by three key features: a dense, polyclonal lymphoplasmacytic infiltrate rich in IgG4-positive plasma cells, fibrosis with at least focal storiform architecture, and obliterative phlebitis, where venous channels are destroyed by the inflammatory infiltrate. Elastin staining can assist in identifying fully obliterated vessels [15].

The diagnostic threshold for IgG4+ plasma cells varies by tissue type, for example, >10 cells per HPF in the meninges versus >100/HPF in skin. Regardless of the site, an IgG4+/IgG+ plasma cell ratio >40% is generally considered significant [6,15].

Recent advancements have highlighted flow cytometry of plasmablasts (CD19+CD20−CD27+CD38+) as a sensitive diagnostic tool for untreated IgG4-RD, with a sensitivity of 95% and specificity of 82% [16]. According to the 2019 classification criteria, while serum IgG4 levels can aid diagnosis and monitoring, elevated levels are no longer required for diagnosis [17]. Involvement of the meninges or retroperitoneum may not always be accompanied by elevated serum IgG4 levels [17].

MRI is the preferred imaging modality for assessing HP, typically revealing smooth linear or mass-like thickening of the dura over the brain and spinal cord. CT scans may complement MRI by detecting bony involvement.

Treatment of idiopathic HP typically begins with corticosteroids-prednisone at an initial dose of 1 mg/kg/day. If there is inadequate response or relapse, immunosuppressive agents such as azathioprine (2-3 mg/kg/day), methotrexate (20-25 mg/week), cyclophosphamide (500-750 mg/m² every four weeks), or rituximab may be added [18]. Rituximab, a monoclonal antibody targeting CD20, has shown efficacy in patients who are refractory to glucocorticoids or require steroid-sparing therapy due to side effects [19].

## Conclusions

IgG4-RD is a chronic inflammatory condition that can involve various organs, including the central nervous system, where it can manifest as HP. This manifestation is rare but can cause a wide range of neurological symptoms due to fibrotic thickening of the dura mater.

Diagnosis can be challenging and relies on a combination of clinical, radiological, and pathological findings. Since blood tests alone are not definitive, tissue biopsy remains essential. Early recognition and treatment are important to prevent long-term complications and improve outcomes.
